# A Railway Track Geometry Measuring Trolley System Based on Aided INS

**DOI:** 10.3390/s18020538

**Published:** 2018-02-10

**Authors:** Qijin Chen, Xiaoji Niu, Lili Zuo, Tisheng Zhang, Fuqin Xiao, Yi Liu, Jingnan Liu

**Affiliations:** 1 GNSS Research Center, Wuhan University, No. 129 Luoyu Road, Wuhan 430079, China; chenqijin@whu.edu.cn (Q.C.); xjniu@whu.edu.cn (X.N.); jnliu@whu.edu.cn (J.L.); 2 Collaborative Innovation Center of Geospatial Technology, Wuhan University, No. 129 Luoyu Road, Wuhan 430079, China; 3 Wuhan Municipal Construction Group Co., Ltd., Wuhan 430023, China; zll2557325156@163.com (L.Z.); xiaofuqing123@163.com (F.X.); 4 Shenzhen Datie Detecting and Surveying Inc., Shenzhen 518109, China; liuy@szdatie.com

**Keywords:** railway, track geometry surveying, aided INS, track trolley, inertial surveying, mobile surveying

## Abstract

Accurate measurement of the railway track geometry is a task of fundamental importance to ensure the track quality in both the construction phase and the regular maintenance stage. Conventional track geometry measuring trolleys (TGMTs) in combination with classical geodetic surveying apparatus such as total stations alone cannot meet the requirements of measurement accuracy and surveying efficiency at the same time. Accurate and fast track geometry surveying applications call for an innovative surveying method that can measure all or most of the track geometric parameters in short time without interrupting the railway traffic. We provide a novel solution to this problem by integrating an inertial navigation system (INS) with a geodetic surveying apparatus, and design a modular TGMT system based on aided INS, which can be configured according to different surveying tasks including precise adjustment of slab track, providing tamping measurements, measuring track deformation and irregularities, and determination of the track axis. TGMT based on aided INS can operate in mobile surveying mode to significantly improve the surveying efficiency. Key points in the design of the TGMT’s architecture and the data processing concept and workflow are introduced in details, which should benefit subsequent research and provide a reference for the implementation of this kind of TGMT. The surveying performance of proposed TGMT with different configurations is assessed in the track geometry surveying experiments and actual projects.

## 1. Introduction

Railway track geometry quality has a decisive influence on the dynamic performance of the vehicle [1], and railway operating safety and passenger comfort depend to a large extent on the smoothness of the railway track, especially for high-speed railways. The track condition tends to deteriorate due to external factors, such as the frequent passage of heavy trains and deformation of the track bed. These factors make the railway track drift away from its designed geometric position and result in track irregularities. Track irregularity, i.e., track deformation, is one of the most important factors that cause safety problems and further track deterioration, so accurate measurement of the track geometry is a task of fundamental importance for adjusting deformed tracks and ensuing high operational safety.

Railway track geometry is characterized by its external and internal geometric parameters. The external track geometry is defined by the absolute position of the track axis/centerline in 3D space, and the internal geometry is determined with five types of principal parameters, including alignment, longitudinal level, gauge, cross level and twist [2]. There are different accuracy requirements concerning the track smoothness and the absolute position of the track in the geodetic reference frame [3]: (a) For example, the high speed railway track must be maintained with an absolute position accuracy of 1 cm relative to the track geodetic control network and sub-millimeter relative accuracy to control the short wavelength track irregularity and ensure track smoothness and inner geometry consistency; (b) The requirements in terms of track correction values to guide tamping machines or precise adjustment of slab track are about 1 mm; (c) For the norm speed ballasted railway, it is practically required to survey the track axis with 1–5 cm accuracy for redesign, building an adjacent new line or reconstructing the geometric parameters to improve the operating speed of an existing line. Besides these accuracy requirements, time efficiency is another critical issue for track surveying, because the time slots or skylight time permitted for railway surveying each day for an existing line is typically limited due to the high traffic volumes.

The overall railway track geometry condition of the existing line is inspected regularly by dedicated track inspection trains or track recording vehicles [4] owned by the railway management department. Dedicated track inspection trains can survey at high speed, but provide only relative information which hardly conforms to the accuracy requirements in the determination of the track geometric deformation and their related localization for tasks such as precise track adjustment applications. In addition, it is too expensive and inflexible to use heavy dedicated track inspection trains for geometry surveying on short track sections or on construction sites. Lightweight and flexible track geometry measuring trolleys (TGMTs) in combination with high precision geodetic surveying apparatus were developed for geometry measurements of unloaded tracks, and provide follow-up accurate measurements after the dedicated track inspection train for regular maintenance of existing lines and accurate measurement for alignment, precise adjustment or tamping applications during the railway construction stage [5].

There are a variety of TGMTs with different functionalities, handling different tasks and using different surveying technology, which can be roughly classified into two typical categories: the relative measuring trolley/relative TGMTs and absolute measuring trolley/absolute TGMTs, but sometimes it is not easy to distinguish between them.

The relative TGMTs mainly measure part or all of the internal geometric parameters and provide relative measurement information by using some relative measuring techniques such as the Hallade/versine method [1,3,6], inertial sensors, and laser alignment methods [7]. The classical chord method or Hallade surveying originally used in dedicated track inspection trains are adopted in the relative TGMTs to measure the versine of a predefined chord to evaluate the inner geometric consistency of the track. The inertial surveying method typically uses inertial sensors such as gyroscopes or inertial measurement units (IMUs) to measure the track attitude angles, including roll, pitch and azimuth, then the IMU trajectory is derived by integration of the pitch and azimuth angle measurements along the covered distance provided by distance sensors such as odometers. The measurement accuracy of the inertial surveying method degrades as the covered distance increases.

Absolute TGMTs measure the absolute external geometric parameters together with part or all of the internal geometric parameters. Absolute track axis/centerline position coordinates are determined instantly by such absolute geodetic surveying apparatuses as total stations or global navigation satellite system (GNSS) receivers. A number of previous research works on TGMTs in combination with classical geodetic apparatuses can be found, for example the Rhomberg track surveying trolley originally developed by the University of Stuttgart, the SURVER trolley developed by German Railway DB, and the Geo++ trolley of Garbsen. More details on these trolleys can be found in [3]. Galus [3] at ETH Zurich designed a modular multisensory track surveying trolley system, the Swiss trolley, which integrates a total station, laser scanner, cameras and GNSS to determine the track geometric parameters instantly while measuring. Details on the design and functionalities of the TGMTs are introduced thoroughly and comprehensively in his Ph.D. thesis. Akpinar [8] designed a multisensory railway track geometry surveying system based on GNSS, total station, linear variable differential transformers and inclinometer which is used as an alternative to classical geodetic measurement methods to control the railway track geometry. The adaptive Kalman filtering algorithm is adapted for instant determination of the track geometric parameters including track gauge, cross level, gradient and track axis coordinates [9]. More similar literatures can be found in [5,10].

TGMTs using these classical geodetic apparatuses such as total station and GNSS in combination with gauge and cross level measuring sensors are a proven technology for precise railway track geometry surveying, and it is now the instrument manufacturing companies’ business to customize and produce such kind of TGMTs. The most well-known product using this technology is the Amberg GRP surveying system series by Amberg Technologies (Regensdorf-Wattcity, Switzerland), and the Trimble GEDO system by Trimble Inc. (Sunnyvale, CA, USA). Typical absolute TGMTs, such as the Amberg GRP1000, use an autonomous motorized total station to measure the 3D coordinates of the trolley-borne prism relative to the geodetic control network, in which case the total station is typically placed near the track axis and the TGMT stops at the sleeper/fastening system point or at pre-determined distance intervals and remains stationary for static measurements. The TGMT combined with a high precision total station is able to achieve about 1 mm accuracy in this stop-and-go surveying mode when the line of sight is shorter than 70 m. Obviously absolute TGMTs are accurate enough for railway track geometry surveying, but the speed is fair low due to the stop-and-go surveying mode, and only about 150-m track per hour can be surveyed by this method. GNSS is also widely used for absolute geo-referencing to achieve 2–5 cm measurement accuracy in the post-processing kinematic mode.

Is it possible to measure the track geometry with 1 mm absolute accuracy in real mobile surveying mode and significantly improve the speed, for example to 2.5 km/h, compared to the conventional stop-and-go mode (0.15 km/h). It is obvious that both the conventional absolute and relative TGMTs each have their own shortcomings and neither of them can address this problem, so clients have to compromise between the measurement accuracy and surveying speed when choosing a method. Actually, achieving 1 mm absolute accuracy in mobile surveying mode is a big challenge for the whole geodesy and surveying discipline.

There is little previous research work attempting to address this problem from different aspects. The authors of this work proposed in 2013 a novel method of measuring the railway track irregularity based on INS/GNSS integrated system, which is capable of measuring the track irregularity with 1 mm relative accuracy and significantly improve the surveying efficiency, and is the primary prototype of the TGMT based on aided INS introduced in this paper [11,12]. Since then there has been some follow-up research on TGMTs that use the inertial navigation technique to improve the surveying speed. Li [13] used the laser-aided INS/odometer integrated system to measure the subway track irregularity where the geo-reference absolute position relative to the geodetic control network is determined by the laser scanner to estimate the INS drift. Jiang [14,15] introduced a data processing algorithm for TGMTs based on IMUs/odometers and analyzed the theoretical error propagation of the INS/odometer integration updated by landmark and zero velocity. Since 2015, Amberg Technologies has updated their Amberg GRP system by integrating an IMU to improve the surveying efficiency of models such as the Amberg IMS 3000, and Leica Geosystems and Trimble Inc. claim they will offer similar TGMT products in the coming future, but insight into the technical details of these commercial products is limited. Further studies are need to comprehensively address the related technique problems on the TGMTs based on aided INS which aim at achieving 1 mm accuracy in mobile surveying mode.

The contribution of this paper is to provide a comprehensive solution to the challenges mentioned above by integrating INS with a precise geodetic surveying method such as total station and GNSS to provide accurate measurement of the track geometry aiming at different task in mobile surveying modes with a modular system design. Since TGMTs based on aided INS for fast and accurate track geometry surveying are a newly emerging technology, key points of the design of the TGMT’s architecture and the data processing concept and workflow are introduced thoroughly, which should benefit subsequent research and provide a reference for the implementation of this kind of TGMTs based on aided INS. This paper is organized as follows: Section 2 presents the basic measuring principle of the TGMT based on aided INS. Section 3 is devoted to the concept and key points of designing the track geometry measuring trolley system. The concept and workflow of the data post processing is introduced in Section 4. Field test and result analysis is presented in Section 5.

## 2. Measuring Principle of TGMT Based on Aided INS

The rail or track axis is a three dimensional curve by nature and there are different accuracy requirements concerning the rail smoothness and its absolute position in the reference geodetic frame. The railway track geometry can be uniquely determined with the 3D position coordinates of the track axis or reference rail together with gauge and cross level. Once the track position, gauge and cross level are measured accurately, the actual track geometry can be reconstructed, track geometric parameters and deformation can be identified, and track geometric quality can be evaluated. The definition of the principal track geometric parameters including gauge, cross level, alignment, longitudinal level, twist, together with versines with respect to a chord of given length, and track irregularities in both the horizontal and vertical directions can be found in [2,11,12].

The TGMT based on aided INS integrates an IMU, gauge measuring sensor, odometer and absolute positioning sensor on a specific lightweight track trolley to survey the track geometry in kinematic/mobile surveying mode. The track trolley without suspension connection maintains continuous contact with the railway track in both the across-track and vertical directions. The trolley wheels are used to link the aided INS and the rails mechanically, which allows the inertial sensors to “sense” the track geometry and deformation. Railway track irregularity inspection is based on the response of the track trolley to the track irregularities, which would cause a motion state fluctuation of the trolley moving on the rails. These response signal can be captured by the aided INS.

**Figure 1:**
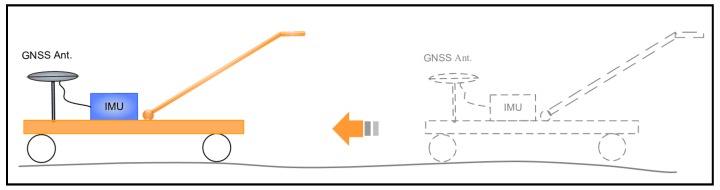
Illustration of the TGMT based on GNSS/INS system.

INS is well known to be capable of providing position and attitude measurements with extremely high relative accuracy in a short time range. Aided INS can take advantage of external update information, such as GNSS or total station position, to correct the navigation error accumulation of the INS so as to maintain its relative measurement accuracy over a longer time range and attach the measurements to an absolute geo-reference. Figure 1 illustrates the measuring principle of the TGMT based on aided INS: the TGMT captures the 3D position, attitude angles, gauge accurately and simultaneously, based on which the track geometry is determined and all principal track geometric parameters can be derived.

## 3. Concept of A-INS Railway Track Measuring System

**Figure 2:**
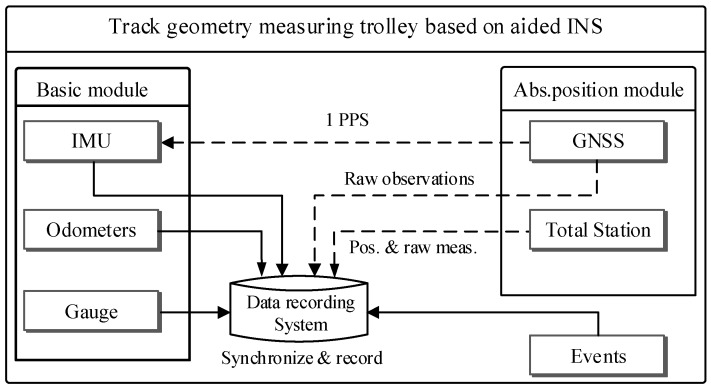
Modular design of the TGMT based on aided INS.

The TGMT based on aided INS is designed to capture accurate 3D position coordinates, gauge, and event signals simultaneously, based on which all railway track geometric parameters can be derived and track quality can be evaluated and associate the precise localization information to the measured data. The TGMT based on aided INS is a multisensory surveying system which integrates several units including track trolley platform, inertial measurement unit, absolute positioning module such as total station or GNSS, gauge measuring sensor, odometer, sleeper recognizer, data recording system. A modular design is adopted for the system’s structure implementation as depicted in Figure 2. The basic and common module contains track trolley platform on which all sensors are fitted: IMU, two odometers, and gauge measuring sensor. The absolute positioning module provides an absolute geo-reference for the multi-sensor data fusion and can be configured as a GNSS or total station module according to different surveying tasks. Event signals are recorded simultaneously to label some important information, such as tagging the position of a tie/sleeper. All data from different sensors are centrally synchronized in time and recorded in the data recording system. Key points of the surveying system and sensors are introduced in the following subsections.

### 3.1. Track Trolley

**Figure 3:**
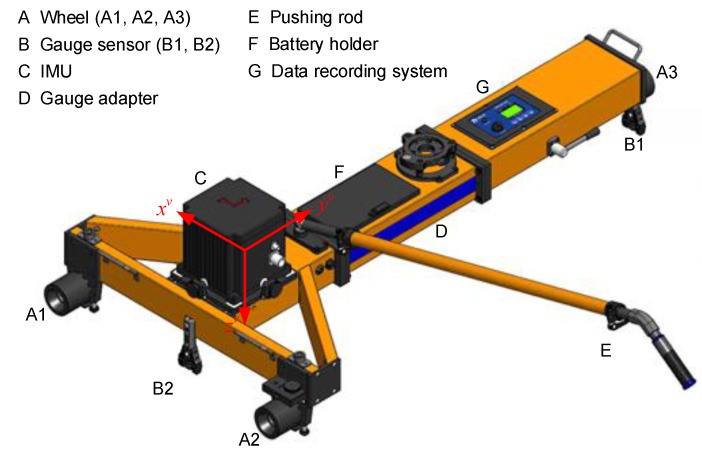
TGMT based on aided INS design (lateral view).

**Figure 4:**
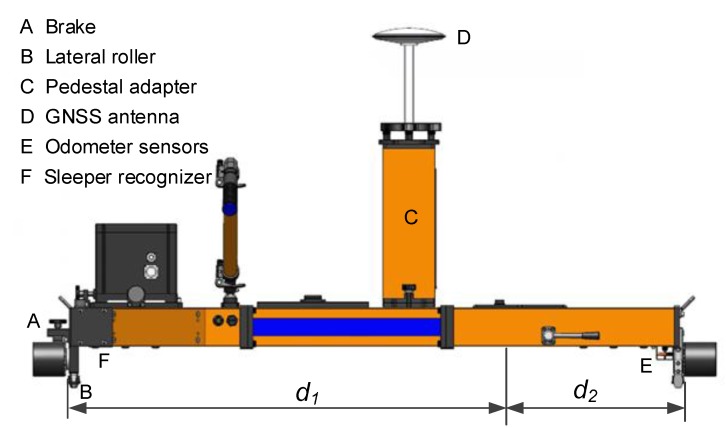
TGMT based on aided INS design (front view).

The rigid and accurate trolley is the platform on which all measuring systems and sensors are fitted. Conforming to the normative railway standards, the trolley is designed as a portable T-shaped structure which does not load the track and can readily be placed on or off the track by two operating surveyors as shown in Figure 3 and Figure 4. The trolley consists of two beams with one along the x-direction and the other along the y-direction. An adapter is designed available for different track gauges. The trolley can be pushed or hauled by human force with a pushing rod and is capable of measuring from standstill to the maximum permissible speed of 5 m/s. A handbrake is adopted to prevent unintentional motion when the trolley must stay at rest for static measuring, such as the static alignment process of the INS and free stationing of the total station. The trolley has three wheels fixed to the platform that contact the rails on the upper surface of the head of the rail, i.e., running table, the axles of which are coplanar and all perpendicular to the x-direction. A lateral roller is mounted under each wheel with its axle coplanar with those of the related wheels. The rollers contact the inner surface of the rails at the gauge measuring point specified by the normative standards. Two rollers on the left hand side, i.e., along the *x*-axis direction under wheel A1 and wheel A2 as depicted in Figure 3, are fixed with trolley platform guiding the motion of the trolley, and the third one is fixed with a gauge measuring spring to form a free expansion end. The acting force of the gauge measuring spring along the y-direction presses the rollers against the inner surface of the rails to ensure rigid and reliable contact is maintained. Adopting the lateral rollers architecture design has advantages over the wheel flange for its longer life span and reduced necessity for maintenance when working in kinematic/mobile surveying mode. The response of the platform to rails’ geometric deformation is the premise of track geometry survey, thus the wheels of the trolley should be able to keep reliable and continuous rigid contact with the rails when moving on rails. This requires that the trolley does not jump off with respect to the rails in the vertical direction and not slide laterally, in which case the track geometry can be derived from the trajectory of the trolley. Special attention should be paid to the magnitude of the acting force of the loaded spring to guarantee the continuous and rigid contact, especially in the case when the wheel A3 is put on the lower rail on the curved track section. A universal pedestal adapter in the center of trolley structure is designed as a holder of a total station, GNSS antenna or prism.

As a multisensory system, different sensors cannot be physically co-located on the host trolley platform, and their mounting positions are distinct, which is known as the lever arm. The lever arm is a three dimensional vector defining the displacement from the origin of the reference frame, to the measurement point of different sensors and usually revolved in the reference frame. All lever arms for different sensors should be measured and calibrated accurately in the identical reference coordinate frame. Within this work, we choose the host vehicle frame as the reference frame. The vehicle frame (v-frame) is rigidly attached to and defined with the host trolley carrying the measuring system and sensors with its origin centered at the IMU center as depicted in Figure 3. The *x*-axis points forward along the vehicle longitudinal direction parallel to the straight line formed by the two contact points of the left rollers with the inner rail surface, the *z*-axis is perpendicular to the plane formed by the contact points of the three wheels and points downward, and the *y*-axis point outward, completing the right-hand set. There are different lever arms those should be accurately measured include the positioning sensor (GNSS, or total station), odometer contact points of the wheels and rollers, the IMU and so on.

### 3.2. IMU

Inertial measurement unit (IMU) is the core sensor of the TGMT based on aided INS, which is used to bridge the positioning gap between two external absolute position from a GNSS or total station. The measurement accuracy differs for different types of IMU, and the choice of IMU depends on the accuracy requirement, surveying task and how frequent the external absolute update information is available. The longer the inertial navigation system is required to work alone the higher the grade of IMU that should be chosen. Since the IMU is mounted on the trolley without any suspension, the IMU is required to be able to withstand some shock and vibration, especially when the trolley is moving at high speed over welded joints. The operating temperature range should also be taken into account since the IMU may be used in a wide temperature range in real railway track surveying. In our design, a navigation grade IMU POS830 manufactured by Wuhan MAP Space Time Navigation Technology Co., Ltd. (shortened as MAP Inc., Wuhan, China) is integrated. It contains a linear quartz accelerometers triad and a ring laser (RLG) gyros triad, and measures acceleration and angular velocity in three mutually orthogonal direction centered at the IMU measuring center, and outputs datasets as incremental velocity and angles. A NovAtel OEMV-2 receiver is built in this system to provide raw GNSS observations at 1 Hz sampling rate. The IMU and GNSS receiver is enclosed in a fully sealed housing designed to withstand water, oil and gas spray. The IMU performance specifications are listed in Table 1.

The coordinates of the IMU measurement center, nominally defined as the intersection point of the built-in accelerometer trials, in the v-frame should be calibrated accurately. In practice a chosen reference point rather than the actual measurement center of the IMU are referred to since the actual measurement center is not easy to measure. The aided INS can provide the attitude measurement of itself including roll, pitch and heading angle, while in the coordinate transformation from the measuring sensor to the reference coordinated frame we need the attitude of the trolley platform. Thus the mounting angles or attitude misalignment of the IMU, i.e., the physical angular offset of the IMU body frame with respect to the vehicle body frame (v-frame), should be measured and calibrated accurately which effects the following coordinate transformation. Uncompensated roll and pitch misalignment will result in constant roll and pitch measurement errors, and consequently will produce constant slope and cross level measurement error. For example, to achieve 0.1 mm accuracy in cross level measurement, the residual misalignment around roll axis after calibration should be smaller than 0.005 deg. In addition, the mounting angles also effect the performance of the non-holonomic constraint (NHC) of the rails on the motion of the trolley in the aided INS algorithm [11,16].

Therefore, it is critical to estimate and calibrate the mounting angles as accurately as possible, and at least as accurate as the attitude measurement of the aided INS. The mounting angles can be estimated through several approaches. The misalignment around the roll and pitch axes can be easily estimated to a relatively high accuracy by comparing the static attitude measurement from the TGMT in the forward-orientated and reverse-orientated at the same mileage point. The misalignment around the heading axis can be computed by differencing the heading measurements and the reference truth in the calibration lab, or by differencing the heading measurements and the nominal track azimuth series derived from the design file of the railway track. Though the IMU does not need to be mounted in particular orientation, in our TGMT the IMU is mounted with the IMU body frame approximately coinciding with the v-frame. Three specific alignment pins are fixed on the trolley platform to ensure the mounting repeatability of the IMU. The misalignment angles around the heading and pitch axis can also be estimated and calibrated in the data processing algorithm with odometer speed and NHC aiding [16,17].

### 3.3. Absolute Positioning Unit

Absolute positioning module in the TGMT based on aided INS is designed to provide absolute geo-reference data such as the absolute position coordinates to estimate and correct the inertial sensor errors, ensure the absolute measurement accuracy, and attach precise localization information to all measurement results. The requirement for absolute measurement accuracy differs for different surveying tasks which will be discussed in Section 5, thus two absolute positioning modules are optional, including a GNSS and total station. The GNSS configuration is designed to provide centimeter level coordinates and a total station is available to achieve an absolute measurement accuracy of 1~2 mm relative to the geodetic control network.

#### 3.3.1. GNSS

GNSS is an important method for providing absolute positions in the global coordinate frame with different accuracy levels in geodetic surveying applications. A post processed carrier-phase-dominated differential GNSS is adapted by the TGMT to provide centimeter level positions. The build-in GNSS receiver in the POS830 works as the rover, and the master GNSS receiver locates at a known position, such as the geodetic control network points of the railway. The rover and master receivers record raw GNSS observations at 1 Hz sampling rate simultaneously, and the baseline length usually does not exceed 15 km to ensure centimeter accuracy.

A GNSS would always be a good option for the measuring trolley system to provide absolute geo-reference data and integrate with INS whenever GNSS signals are available. The benefits and drawbacks of INS and GNSS are complementary, so by integrating them, the advantages of both technologies are combined to give a continuous, high-bandwidth, complete navigation solution with high long- and short-term accuracy. In the GNSS/INS integrated system GNSS measurements prevent the inertial solution from drifting, while the INS smooths the GNSS solution and bridges signal outages [18]. A loosely coupled GNSS/INS integration algorithm that uses the GNSS position and/or velocity solutes as the measurement inputs to the Kalman filter is adapted for the data processing for its simplicity. Carrier-phase-dominated DGNSS/INS provide high frequency positioning solution with centimeter accuracy and much higher relative measurement accuracy.

The TGMT with GNSS/INS module can realize submillimeter relative accuracy in inner track geometric parameter measurement including the track deformation/displacement or track irregularity measurement, however this is not sufficiently accurate for determining the absolute position of the track when millimeter absolute positioning accuracy is required. Therefore, the available application scenario of the TGMT with GNSS/INS module is restricted which will be discussed in details in Section 5.

#### 3.3.2. Total Station

**Figure 5:**
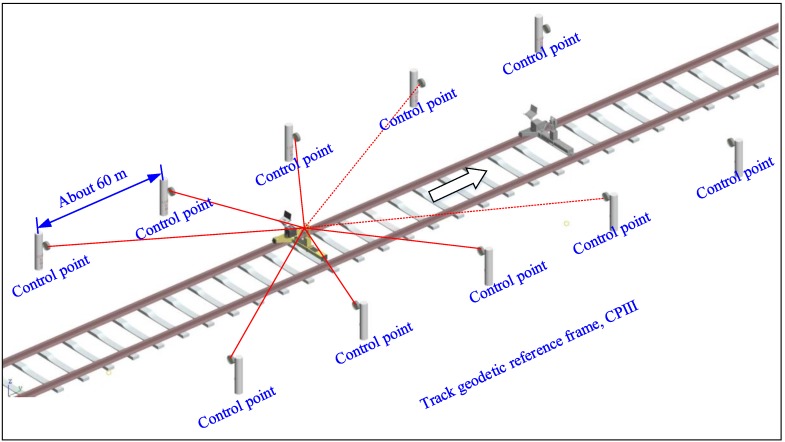
Free stationing procedure.

A high precision total station is an important engineering surveying apparatus that provides absolute geo-reference data with submillimeter accuracy relative to the track geodetic control network. The total station is designed to be fixed on the host trolley and moves with the TGMT. The advantage of mounting the total station on the moving host trolley platform rather than on an outside tripod near the track axis is the improvement of stationing efficiency, since the total station can be transferred to next stationing site and set up quickly and the total station does not need leveling before stationing. The TGMT stops and keeps in rest at each point where a total station measurement should be carried out. The location of the total station in the reference coordinate system of the control network is determined by a free stationing method, i.e., resection. During the free stationing process, bearings and distances of the total station to 5~8 known back-sight points of the railway geodetic control network surrounding the stationing site are measured, as illustrated in Figure 5. The local polar coordinates of the back-sight points defined by the horizontal circle of the total station can be readily calculated. With a similarity transformation, these local polar coordinates are transformed to the coordinates system of the track control network, and errors are distributed by least squares adjustment. The position and attitude angles of the total station in relation to the control network is determined.

**Figure 6:**
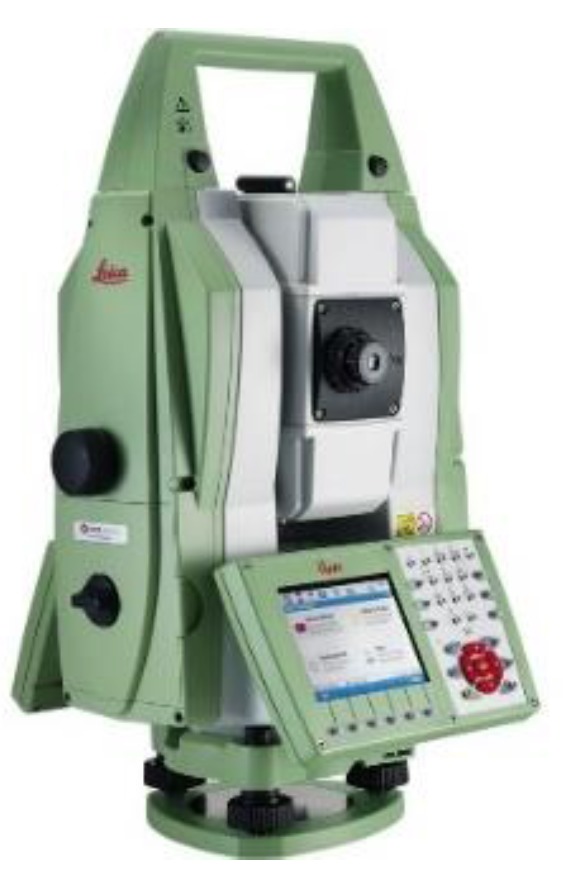
Leica TS16 total station.

Measurement accuracy is the first consideration in choosing a total station. Special attention should be paid to protect the total station from mechanical shock when experiencing large accelerations while moving over features such as weld joints. For the TGMT based on aided INS, a Leica Nova TS50 optical tracking total station is integrated. The Leica Nova TS50 (Leica Geosystem AG, Heerbrugg, Switzerland), shown in Figure 6, is the latest generation of autonomous tracking total station with 0.5 automatic aiming accuracy, and 0.6 mm + 1 ppm to prism electronic distance measurement accuracy. By measuring 5–8 redundant back-sight control points, submillimeter stationing accuracy can be ensured. The free stationing provides submillimeter position to integrate with the INS to give a high frequency coordinate measurement. For accurate integration, the lever arm of the total station measurement center in the host vehicle frame, and the mounting angles of the total station relative to the IMU should be measured and calibrated. The total station measurements are synchronized and recorded centrally in the data recording system.

About 1.4 mm positioning accuracy of the TGMT with TS/INS module can be achieved when the free stationing of the total station is carried out every 120 m which will be validated in Section 6.2, and longer distance interval between two adjacent stations will lead to an accuracy degradation.

### 3.4. Gauge Measuring Sensor

Track gauge is measured using a high-precision contact mechanical distance measuring system. The gauge measuring sensor is by nature a distance measurement sensor fixed on the beam of the trolley which is perpendicular to the *x*-axis of the vehicle frame, and measures the length variation of the loaded spring. During the surveying process the spring is under compression, thus the spring presses the three rollers to the inner surface of both rails to keep reliable and continuous contact with rails.

**Figure 7:**
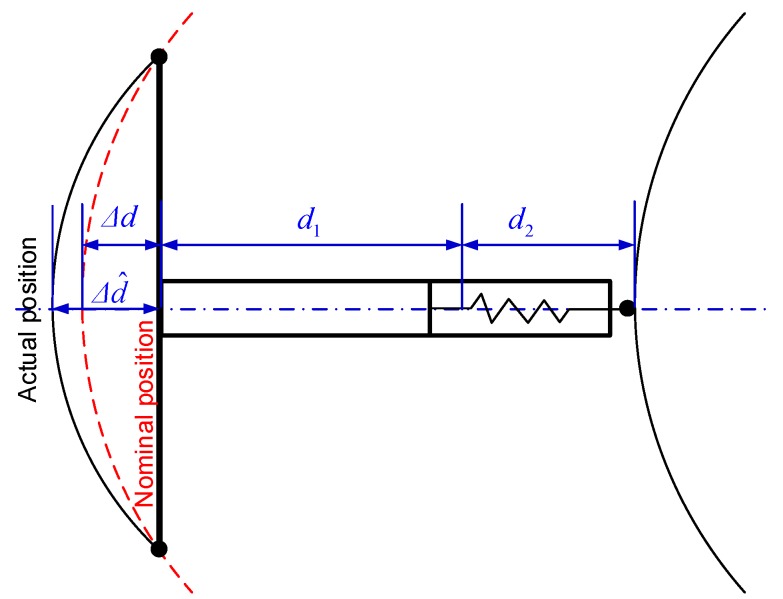
Gauge measurement compensation in the curved track section.

The gauge measurement as depicted in Figure 7 can be represented as:

(1) $$d=d_{1}+d_{2}+\Delta d+K_{T}T$$

where *d* is the gauge measurement, *d*_1_ is a constant offset, *d*_2_ represents the varying part of the gauge, *K_T_T* is the correction term induced by temperature variation, the related coefficient *K_T_* can be calibrated and *T* is the temperature value measured by thermometer. *Δd* is the gauge correction term induced in the curved track section; its theoretical value is computed as:

(2) $$\left|\Delta d\right|=R-\sqrt{R^{2}-d_{w}^{2}/4}$$

where *R* is the radius of curvature of the nominal track axis, *d_w_* is the distance between the axles of wheel A1 and A2 as illustrated in Figure 3. The sign of *Δd* is determined by the turn direction of the curve and the orientation of the trolley set on the rails. It is obvious that *Δd* shall be zero in a straight line section. The larger the radii of curvature is the smaller the *Δd* would be. For example the high speed railway track is usually designed with large radii, in which case the *Δd* is small, while for the rails with small radii such as subway and tramway lines this correction term cannot be ignored. It should be pointed out that *Δd* is just the nominal correction, while the actual value of this term may be slightly different from the nominal value due to the short wavelength track deformation. Thus it is optimal choice to add another gauge measuring sensor between the two wheels along the *x*-axis of the trolley to give the complete measurement of the gauge as depicted in Figure 3.

The gauge sensor shall sample at a sufficiently high data rate when the TGMT based on A-INS is working in kinematic/mobile mode. For example, if the trolley moves at a speed of 5 m/s, and the gauge sensor samples at 20 Hz, then the distance interval of the gauge measuring samples would be 0.25 m. In addition, gauge measurement should be synchronized with IMU data. TGMT based on aided INS is able to measure the absolute track gauge with 0.1 mm accuracy after calibration.

### 3.5. Odometer

The Hall-effect wheel transducer is chosen as the odometer sensor in the TGMT to measure the longitudinal distance travelled by the trolley along rails by counting the number of rotations of the wheels. The distance measured by the odometer sensor contains a cumulative error which is proportional to the longitudinal travel distance due to the uncertainty in wheel radius, resolution of the odometer, dirt adhered to the wheels, and possible slippage. To obtain more reliable travel distance recordings, we mount odometer sensor on both wheel #A1 and #A3 as shown in Figure 3. Odometer measurements from the wheel #A1 and #A3 is combined to give a more robust odometer measurement whose cumulative error versus the travel distance is controlled to be smaller than 0.05%. Two Hall-effect transducers are installed on both wheel #A1 and #A3 in a specific manner, as an effect of the phase shifted signals, the moving directions of the trolley could be derived.

Velocity derived from longitudinal distance measurement is an important auxiliary information and constraint for the inertial navigation, which can effectively improve the accuracy of the integrated navigation solution especially when GNSS measurement is unavailable. Complementary to the non-holonomic constraint, the odometer signal can be regarded as the velocity update along the forward (along-track) direction. Therefore, the non-holonomic constraint and the odometer speed compose a complete three dimensional velocity constraint in the vehicle frame [11,16]. In the aided INS algorithm, the scale factor error of the odometer measurement should be taken into account and augmented into the state vector of the Kalman filter for data fusion, which would be observable when external positioning aiding such as GNSS or position from total station is available. In practice, the lever arm from the IMU measurement center to the contact point of the wheels where odometer sensors are mounted with the rails should be accurately measured and taken into account in the data fusion. Time tags are attached to the odometer sensor recordings so as to synchronize with the IMU raw data. Odometer measurement is also used to aid the zero velocity detection of the TGMT.

### 3.6. Sleeper Detector

Since the correction or precise adjustment of track slabs can be only carried out through the adjustable fastening system, the surveying trolley should output measurements data at the localization of each sleeper or fastening system point. The TGMT based on aided INS is designed for kinematic surveying, which means the trolley does not stop at each sleeper point to measure as the conventional track trolley based on total station such as the Amberg GRP1000 does. Therefore, a TGMT for kinematic surveying should detect and measure the localization of the sleepers, which is implemented by recording the time pulse when the TGMT passes over the sleeper or fastening system.

A reflective ultrasonic sensor is integrated under the trolley beam to detect the sleepers as illustrated in Figure 4. The ultrasonic sensor sends ultrasonic waves from the emitter on the trolley toward the track slab, then receives the reflected waves with a detector. It measures distance between the sensor and the sensing objective, i.e., track slab. The distance between the sensor and the track slab varies significantly when passing over a sleeper, and this event indicating a sleeper would be recorded and synchronized with other sensors. The sleeper detector has the probability of missed and false detection, but this can be identified and corrected easily with the aiding from odometer measurements. Finally, the sleeper localizations are determined by reading the positioning solution through time synchronization information.

### 3.7. Time Synchronization

A kinematic/mobile multisensory system requires accurate time synchronization between the measurements from different sensors. The faster the TGMT moves the more accurate the time synchronization that should be carried out. Since the multiple sensors on the TGMT have different data sampling rates, the event-driven method [3] is adopted for time synchronization: all sensor measurements are recorded in a same data recording system, and tagged by the local clock inside the recording system in the identical time frame. Whenever the recording system receives a measurement signal from sensors, the local time tag is insert into the data package and then stored in the system. The local time provided by the data recording system may drift due to the frequency instability but does not effects the mutual time synchronization between sensors. When GNSS is available the local clock drift can be checked and corrected by introducing the 1 PPS signal (pulse per second) from the GNSS receiver, and the identical local time system can be transformed to the more accurate and uniform GNSS time system.

**Figure 8:**
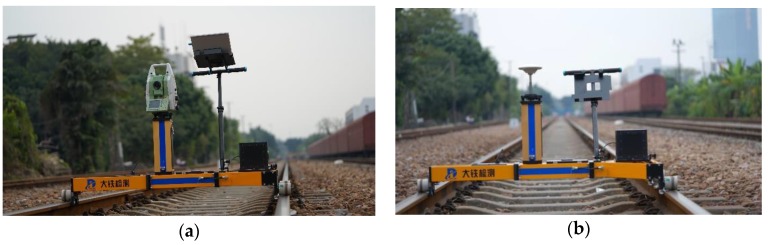
Modular design of the TGMT based on aided INS. (**a**) TGMT with TS/INS configuration; (**b**) TGMT with GNSS/INS configuration.

The time synchronization for aided INS integration is critical. When the GNSS/INS module is adopted as shown in Figure 8a, the accurate time synchronization can be ensured from the high precision GNSS time. In the case when total station is configured as an external absolute positioning sensor as shown in Figure 8b, the time synchronization between IMU and total station is easy since the TGMT is keep static when total station measurements are carried out. The time synchronization between the positioning sensor and the gauge, sleeper recognizer measurement only effects the localization accuracy along the mileage direction where centimeter synchronization accuracy in distance is sufficient and can be easily realized. For example, when the TGMT moves at a speed of 5 m/s, 5 cm synchronization accuracy in mileage can be guaranteed with time synchronization accuracy of 10 ms.

## 4. Data Processing Concepts

**Figure 9:**
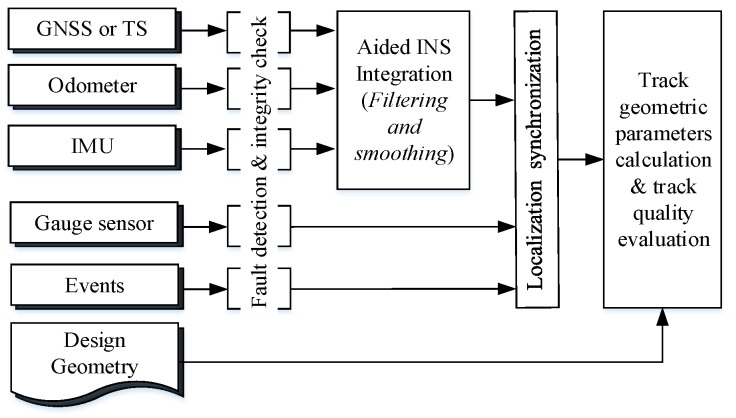
Concept of data post processing.

The synchronized datasets from multiple sensors will be dealt with centrally with a post-processing software to produce the final solutions. In this section the concept and workflow of the data processing is introduced, and key points of the algorithm are emphasized, while the details will be given in a future companion paper. The post-processing software adopts a cascading design which means measurements from different sensors will be preprocessed prior to being fed into the data fusion Kalman filter as shown in Figure 9. This loosely coupled architecture allows for a flexible and modular processing. The data post-processing software contains five modules including: (1) pre-processing, include data integrity check, detection and exclusion. (2) positioning process of the GNSS or the total station. (3) aided INS processing, which includes Kalman filtering and smoothing. (4) mileage/spatial synchronization of all measurements from different sensors; (5) track geometric parameter calculation and track quality assessment. Finally, the implementation of the data post processing software will be introduced.

### 4.1. Data Fault Detection and Integrity Check

Like any technology or system, the TGMT based on aided INS are subject to hardware or software failures, and can occasionally outputs outliers much larger than the uncertainty bounds. Integrity checking module detects and isolate these faults in each channel and protect the final measurement solution from being contaminated [18]. Data integrity checks are carried out mostly in the pre-processing phase for each sensor prior to being sent to the data fusion filter.

IMU measurement integrity should be checked including the IMU measurement failures, sampling gaps and the sensor measurement errors such as those due to shock and variation, and sensor biases. Individual inertial sensor faults are due to hardware failures and can manifest as no outputs at all, null readings, repeated readings, or simply much larger errors than specified [18]. Small sampling gaps arising in the raw data can be accepted and recovered by interpolation to fill in the sampling gap. However, once a large gap has been detected, such as more than three successive missed epochs, the data after the large gap should be discarded. Special attention should be paid to the time synchronization between the ring laser gyro IMU and the GNSS when the inner FIR filter is implemented for the raw gyros’ measurements which may introduce uncompensated time latency and degrade the navigation accuracy of the GNSS/INS integrated system.

Outliers or unexpected signals manifesting as isolated impulsive spike signals can occasionally be present in the gauge, odometer, attitude angle measurements and positioning solutions, especially when the trolley passes over a weld joint or experiences some unexpected large acceleration or vibration. These impulsive spike signals should be detected and filtered to prevent them from being fed into the following data processing. The measurement of the gauge, attitude angle measurement can be treated as one dimensional signal, the impulsive spike signals can be detected simply with a median filter [3]. The outliers in GNSS or total station positioning solutions can be detected dealt with through the innovation filtering in the data fusion Kalman filtering which will be discussed later.

### 4.2. Positioning Process

If GNSS is adopted as the absolute positioning sensor, the post processed carrier-phase-dominated differential GNSS will be applied to provide centimeter-level accuracy position. The GNSS positioning engine is developed by the navigation group at GNSS Research Center at Wuhan University cooperated with MAP Inc. The GNSS positioning modular supports standard positioning processing (SPP), differential GNSS, and carrier-phase based differential positioning processing.

Attention should be paid to the fact that incorrect determination of carrier phase ambiguity happens in the complicated railway surveying environment which may result in a several decimeter or several meter errors in GNSS positioning solutions. The total station positioning is also subject to the phase ambiguity resolution problem. Innovation filtering, which also known as spike filtering, measurement gating, is applied in the data fusion Kalman filtering phase prior to the computation of the Kalman gain to detect the outlier from the positioning sensors including GNSS or total station by comparing the magnitude of each normalized measurement innovation with a threshold and rejects the measurement for that iteration where threshold is exceeded.

### 4.3. Aided INS Processing

**Figure 10:**
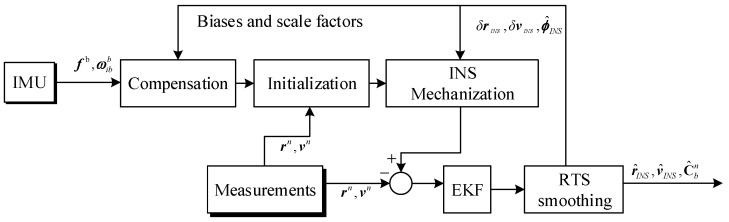
Diagram of the aided INS algorithm in loosely coupled manner.

Fusion of the external auxiliary information and the IMU raw measurement is implemented in a loosely coupled architecture due to the simplicity of its algorithm structure. As the basis of the aided INS processing module, the extended Kalman filter with 22-dimensional error states vectors including 3-position errors, 3-velocity errors, 3-attitude errors, residual biases and scale factor errors of the gyroscopes and accelerometers, and odometer scale factor error is adopted. Moreover, a Rauch-Tung-Striebel (RTS) backward smoothing algorithm is applied in the software to achieve high accuracy in the final solutions. The flowchart of the aided INS integration is depicted in Figure 10 including three main sub-modules: INS processing, Kalman filtering, RTS smoothing.

Outputs of the inertial sensors, i.e., gyroscopes and accelerometers, should be corrected with the sensor errors including biases and scale factors before being fed to the navigation algorithm. The raw IMU measurements is compensated online with the optimally estimated biases and scale factors from the integrated Kalman filter.

Navigation initialization refers to the procedure of determining system’s initial position, velocity and attitude at the beginning of INS processing prior to the kinematic surveying. The initial position and velocity of the INS is obtained from the external positioning sensors, such as the GNSS or total station. The navigation system’s initial attitudes are established through the ground alignment procedure includes two stages for the high grade IMU. The initial phase, coarse alignment of leveling, provides an estimate of initial attitude. In the final phase, fine-alignment, a Kalman filter is used to refine the alignment and estimate inertial sensor errors prior to kinematic surveying [19]. While coarse and fine alignment requires the IMU be of sufficient accuracy to yield a reasonable estimate of initial attitude. If conditions for ground self-alignment does not exist, for example when low grade IMU is used or without sufficient stationary time range, the initial attitudes including bank angle, gradient of slope, and azimuth angles can be derived from the nominal railway track geometry file.

INS mechanization refers to the procedure of updating the navigation results through integrating the IMU measurements, where the compensated accelerometer outputs are rotated and integrated to update the INS velocity and position, and the compensated gyro measurements are integrated to obtain the attitudes [20]. The aided INS system combines navigation state data from INS mechanization with independent redundant external auxiliary data in an extended Kalman filter algorithm, and followed by a RTS smoothing for the post processing mode to benefit from the past and future corrections of adjacent measurement. Dead reckoning based on the aided attitude solutions and odometer measurements can also be adopted to provide the accurate 3D coordinates measurements [14]. The estimated gyros and accelerometers biases and residual scale factors are fed back to compensate the raw IMU measurements, meanwhile the estimated position, velocity and attitude errors are fed back to correct the INS-indicated navigation state data. The final optimal estimation of the total navigation states of the aided INS is produced and stored. The aided INS algorithm also supports other auxiliary information for INS such as zero velocity update (ZUPT), odometer measurements, heading measurements, non-holonomic constraints to improve the aided INS performance. The related algorithm details can be found in [11,20,21].

Since the auxiliary measurement for the aided INS system can occasionally output errors larger than the uncertainty bounds, such as the wrong ambiguity fixing of the GNSS RTK processing may cause a position jump. To protect the overall navigation solutions from contamination of the outliers, fault detection and integrity monitoring strategy based on innovation filtering is adopted in the integration algorithm. The measurement innovations provide an indication of whether the measurements and state estimates are consistent. Innovation filtering may be used to detect large discrepancies immediately, while innovation sequence monitoring enables smaller discrepancies to be detected over time. Details on this algorithm can be found in literature [18].

The integrated algorithm of the aided INS for loosely coupled architecture is introduced briefly below.

#### 4.3.1. System Model of aided INS

A state propagation model is developed here for a Kalman filter estimating attitude, velocity, and position error referenced to earth frame and resolved in local navigation frame (ref to [19]), together with the biases and scale factor of the accelerometers and gyros, and odometer scale factor. The error state vector is written as:

(3) $$x\left(t\right)={\left[\begin{array}{ccccccc} {\left(\delta r^{n}\right)}^{T} & {\left(\delta v^{n}\right)}^{T} & \phi^{T} & b_{g}^{T} & b_{a}^{T} & s_{g}^{T} & \begin{array}{cc} s_{a}^{T} & s_{\mathrm{odo}} \end{array} \end{array}\right]}^{T}$$

where operator *δ* denotes the error of a variable, *δ**r**^n^* = [*δr_N_ δr_E_ δr_D_*]^T^ is the INS-indicated position error resolved in n-frame. *δ**ν**^n^* = [*δν_N_ δ*ν*_E_ δ*ν*_D_*]^T^ is the INS-indicated velocity error resolved in the n-frame. ***φ*** = [*φ_roll_ φ_pitch_* *φ_yaw_*]^T^, the elements *φ_roll_*, *φ_pitch_* correspond to the attitude errors with respect to the vertical, the level or tilt errors, while *φ_yaw_* represents the error about vertical, the heading or azimuth error; ***b*_g_**_,_ ***b****_a_*, ***s****_g_*, ***s****_a_* are dominant inertial sensor residual errors which will be discussed later, *s_odo_* is the odometer scale factor error. The system model in continuous time form is expressed as:

(4) $$\dot{x}\left(t\right)=F\left(t\right)x\left(t\right)+G\left(t\right)w\left(t\right)$$

To obtain the aided INS system model, time derivative of each state variable must be calculated. The position, velocity and attitude error differential equations is [22]:

(5) $$\begin{array}{l} \delta {\dot{r}}^{n}=F_{\mathrm{rr}}\delta r^{n}+F_{\mathrm{rv}}\delta v^{n}\\ \delta {\dot{v}}^{n}=\;C_{b}^{n}\delta f^{b}+f^{n}\times \phi -\left(2\omega_{\mathrm{ie}}^{n}+\omega_{\mathrm{en}}^{n}\right)\times \delta v^{n}+v^{n}\times \left(2\delta \omega_{\mathrm{ie}}^{n}+\delta \omega_{\mathrm{en}}^{n}\right)+\delta g_{l}^{n}\;\\ \;\phi \text{ ˙}=F_{\phi r}\delta r^{n}+F_{\phi v}\delta v^{n}-\omega_{\mathrm{in}}^{n}\times \phi -C_{b}^{n}\delta \omega_{\mathrm{ib}}^{b} \end{array}$$

Symbols in above equations are defined as follows: ***ν****^n^* = [*ν_N_* ν*_E_* ν*_D_*]^T^ is the velocity in the n-frame, *ϕ* and h not in equations are latitude and ellipsoidal height position component, respectively. The radii of curvature along lines of constant longitude and latitude are defined as *R_M_* and *R_N_*, respectively. ***ω****_e_* is the magnitude of the rotation rate of the Earth. *δ**f**^b^* refers to the accelerometer measurement errors, $\delta \omega_{ib}^{b}$ is the gyros measurement errors; $C_{b}^{n}$ represents the b-frame to n-frame transformation matrix. ***f****^n^* is the specific force resolved in n-frame. $\omega_{ie}^{n}$ is the angular rate of the Earth frame relative to the inertial frame in n-frame. $\omega_{en}^{n}\;$is the angular rate of n-frame with respect to the Earth frame resolved in n-frame. $\delta g_{l}^{n}$ is the local gravity error in n-frame. The error rotation vectors $\delta \omega_{ie}^{n}$ and $\delta \omega_{en}^{n}$ are functions of velocity and position perturbations, respectively, and are required to complete the expression for the velocity error equation. The detailed expression of Equation (5) can be found in a number of references [19,20,22,23].

#### 4.3.2. Measurement Model

The navigation aids include GNSS position solutions and vehicle frame velocity (e.g., non-holonomic constraint and odometer) will be considered.

● GNSS Position update

The position of GNSS antenna is related to the INS position by taking into account the lever arm as follows:

(6) $$r_{\mathrm{GNSS}}^{n}=r_{\mathrm{IMU}}^{n}+D_{R}^{-1}C_{b}^{n}l_{\mathrm{GNSS}}^{b}$$

(7) $$D_{R}^{-1}=\mathrm{diag}\left({\left[\begin{array}{ccc} \frac{1}{R_{M}+h} & \frac{1}{\left(R_{N}+h\right)\cos\varphi } & -1 \end{array}\right]}^{T}\right)$$

where $r_{GNSS}^{n}$ and $r_{IMU}^{n}$ are the positions of the GNSS antenna phase center and the IMU measurement center, respectively; and $l_{GNSS}^{b}$ is the lever arm from the IMU to GNSS antenna resolved about the IMU b-frame (ref to [11]) which is assumed here to be well known. $D_{R}^{-1}$ refers to the Cartesian-to-curvilinear position change transformation matrix.

For the loosely-coupled integration, the measurement innovation vector comprises the difference between the GNSS and the corrected inertial position, accounting for the lever-arm effect from the INS to the GNSS antenna. The measurement equation can be derived by perturbation, as:

(8) $$\begin{array}{l} z_{\mathrm{rGNSS}}={\hat{D}}_{R}\left({\hat{r}}_{\mathrm{GNSS}}^{n}-{\tilde{r}}_{\mathrm{GNSS}}^{n}\right)\\ \;\approx \delta r^{n}+\left(C_{b}^{n}l_{\mathrm{GNSS}}^{b}\times \right)\phi +n_{\mathrm{rG}} \end{array}$$

where ${\hat{r}}_{GNSS}^{n}$ is the estimated position of the GNSS antenna center. ${\tilde{r}}_{\mathrm{GNSS}}^{n}$ is the position provided by GNSS receiver. ***n****_rG_* represents the GNSS position error in meter modeled as Gaussian white noise, which is adequate if the GNSS sampling rate is below 1 Hz [23].

● Velocity update in vehicle frame

The aided INS is mounted on a specific track geometry surveying trolley for the railway track surveying applications [11], and the relationship between the trolley wheel velocity and the IMU velocity can be expressed as:

(9) $$v_{\mathrm{wheel}}^{v}=C_{b}^{v}C_{n}^{b}v^{n}+C_{b}^{v}\left(\omega_{\mathrm{nb}}^{b}\times \right)l_{\mathrm{wheel}}^{b}$$

where $l_{wheel}^{b}$ is the lever-arm vector from IMU measurement center to the wheel sensor, resolved in the b-frame. The estimated velocity at the wheel point is denoted as ${\hat{v}}_{wheel}^{v}$.

For the railway track surveying application, the wheels of the track trolley carrying the aided INS are designed able to keep reliable and continuous rigid contact with the rails when moving on rails. Thus the motion of the track trolley on the rails is governed by two non-holonomic constraints (NHC), since the trolley does not jump off and sideslip on the rails. In this case, the trolley has only an along-track speed, and velocities in both cross-track directions are zero. Non-holonomic constraint is considered as navigation aid for the aided INS. The v-frame velocity measurement can also be expressed as:

(10) $${\tilde{v}}_{\mathrm{wheel}}^{v}=v_{\mathrm{wheel}}^{v}-n_{\mathrm{vW}}$$

(11) $${\tilde{v}}_{\mathrm{wheel}}^{v}={\left[\begin{array}{ccc} v_{\mathrm{odo}} & 0 & 0 \end{array}\right]}^{T}$$

where ${\tilde{v}}_{\mathrm{wheel}}^{v}$ is the velocity measurement vector resolved in v-frame, *ν_odo_* represents the along-track velocity derived from the odometer output, ***n****_νW_* is the velocity measurement noise, modeled as Gaussian white noise. The noise strength of the last component of ***n****_νW_* is different from that for the first component and should be set according to the NHC condition.

Therefore, the v-frame velocity error measurement equation can be expressed as:

(12) $$\begin{array}{l} z_{\mathrm{vW}}\;=\;{\hat{v}}_{\mathrm{wheel}}^{v}-{\tilde{v}}_{\mathrm{wheel}}^{v}\\ \;=C_{b}^{v}C_{n}^{b}\delta v^{n}-C_{b}^{v}C_{n}^{b}\left(v^{n}\times \right)\phi -C_{b}^{v}\left(l_{\mathrm{wheel}}^{b}\times \right)\delta \omega_{\mathrm{ib}}^{b}+n_{\mathrm{vW}} \end{array}$$

The direction cosine matrix $C_{b}^{v}$ is assumed to contain no error, since the three misalignment angles between b-frame and v-frame can be well calibrated or measured.

● Smoothing algorithm

A smoothing algorithm is applied to obtain an optimal estimation utilizing all the past, current and future measurements, since the data is allowed to post-processed for accuracy improvement. In this paper, the well-known Rauch-Tung-Striebel (RTS) algorithm is implemented as follows [22]:

(13) $$\begin{array}{l} {\hat{x}}_{k/N}={\hat{x}}_{k}+A_{k}\left({\hat{x}}_{k+1/N}-{\hat{x}}_{k+1}^{-}\right)\\ P_{k/N}=P_{k}+A_{k}\left(P_{k/N}-P_{k+1}^{-}\right)A_{k}{}^{T}\\ A_{k}=P_{k}\Phi_{k}^{T}{\left(P_{k+1}^{-}\right)}^{-1} \end{array}$$

where, **A***_k_* is the smoothing gain, **Φ** is the state transition matrix. **P** is the state error covariance, and subscript *N* is the total number of measurements.

### 4.4. Mileage Synchronization and Resampling

In practice, the aided INS outputs the positioning solutions as the geodetic coordinates, i.e., latitude, longitude, and ellipsoid height, in the Earth-Centered Earth-Fixed frame, while the railway track position is defined in a specific mapping projection coordinate frame. The projected coordinates can be calculated through the Gauss-Kruger projection [24,25], which is an ellipsoid form of the transverse Mercator projection in the first phase. Then the projected coordinates in the Gauss-Kruge plane are transformed to the specific local mapping coordinate frame for the railway track construction through a similarity transformation with a rotation and scale factor estimation, such as the Helmert transformation, with at least two common control points with known coordinates in these two coordinate system [26]. The difference in the height datum between the INS-indicated ellipsoid height and the orthometric height can be determined from the common height control points with height values in these two height datums.

**Figure 11:**
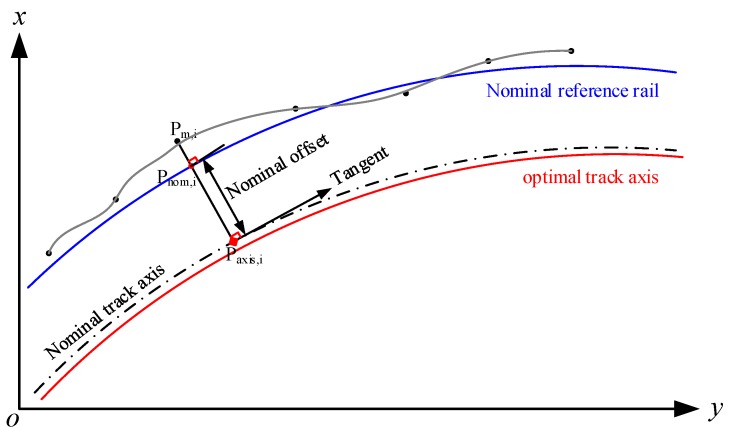
Deviation of alignment.

The localization of the measurement, i.e., mileage along the track, can be determined based on the projected coordinates by deriving the projection of the projected coordinates to the nominal track axis as depicted in Figure 11. All sensors’ measurement can be synchronized with the aided INS positioning solutions through the identical time tagging information.

The IMU, gauge sensors record the raw measurement at high frequency, such as the aided INS provides position solutions at 200 Hz and gauge sensors sample at 20 Hz, which results in a large amount of redundant measurement information. For example, if the trolley surveys at a constant speed of 5 m/s, the aided INS outputs navigation solutions with about 2.5 m distance intervals. For practical railway surveying and precise track adjustment, such dense measurement points are not necessary. Thus the multi sensor measurement should be resampled with identical specific distance interval.

A simple moving average is applied to the measurements series of gauge, height, attitude angles to resample them at a fixed distance interval and smooth out the short-term fluctuation or high frequency noise. The subset size for the moving average is fixed and there is overlapping between adjacent subsets. Robust moving least square fitting is used with the projected coordinates series to keep the inherent relationship between the coordinates in easting and northing [27].

### 4.5. Calculating the Track Geometric Parameters

All principal track geometric parameters can be calculated with the 3D position coordinates, gauge, and cross level measurements. The deviation of gauge and cross level is readily computed by subtracting the designed nominal value from the corresponding measurements. For the TGMT based on aided INS, the cross level is determined by the roll angle measurement from the aided INS. Twist is computed as the algebraic difference between two cross level taken at a defined distance apart [2,11].

Track deviation/deformation in across-track and vertical directions, i.e., alignment and longitudinal level directions, refers to the displacement of the actual rails from their nominal position as shown in Figure 11. It is obtained by calculating the distance from the measuring point on rails to the nominal axis of the track and subtracting the nominal value. Figure 11 depicts the calculation of the alignment, i.e., across-track/horizontal track deformation. If no nominal geometry is defined, such as the case for the old existing line, the track deviation can be expressed as the excursion of measurement point on rails from an optimal axis. The optimal axis can be found with a total least square fitting of 3D position coordinates of the actual rails [2,11,27], or by fitting a curve in the path-tangent angle chart. The principle of the total least square fitting is finding a new optimum trajectory graphically by minimizing the deviation from the fitting curve. The track deviation/displacement in lateral and vertical direction is used for precise track adjustment of the slab track and tamping of the ballast track.

Track irregularity in both the lateral (alignment direction) and vertical direction is defined as the algebraic difference in versines deviations of two measurement points with a defined distance apart. The versine magnitude is distance from the rails to the prescribed moving chord with given length, usually 30 m for the short wavelength, and 300 m for the long wavelength track irregularity [11]. The versines for a prescribed chord is calculated based on the 3D position measurements rather than measured with a real chord method/versine system. Track irregularity indicates whether and where the track should be adjusted, more details can be found in [11].

### 4.6. Implementation of the Data Post Processing Software

**Figure 12:**
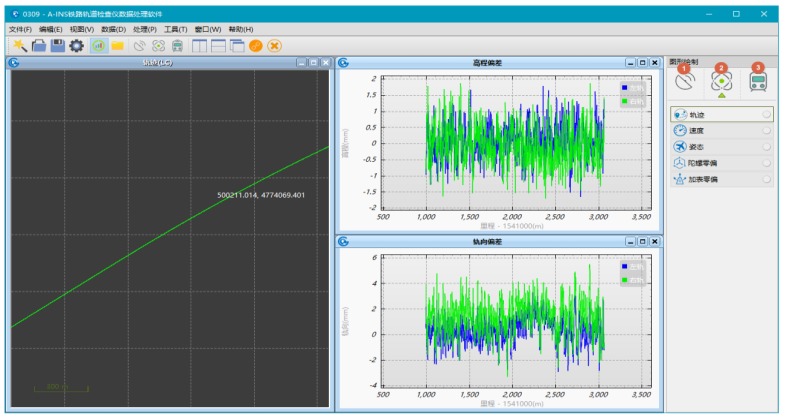
Graphical user interface of the post processing software—*InsRail.*

The data post processing software, InsRail, is developed by the author at the Navigation group of GNSS Research Center at Wuhan University in C++ programming language for the Windows operating system and later customized in cooperation with MAP Inc. as shown in Figure 12. The whole data processing can be completed with InsRail in several minutes immediately alongside the track on the surveying worksite. InsRail is designed able to: (1) create 3D position coordinates along rails and track axis/centerlines, attitude angle measurements including roll, pitch and azimuth, and mileage measurement; (2) calculate all kinds of track geometric parameters including rail deformation magnitude in both horizontal and vertical directions, alignment and longitudinal irregularities, cross level, gauge and twist et al.; (3) associate precise localization information to the measured data and track geometric parameters; (4) produce graphical drawings of the surveying solutions. The surveying reports exported from the InsRail can be used for track geometry quality monitoring, precise track adjustment, and be sent to the tamping machines in industry standard formats.

## 5. Application Scenario

The TGMT based on aided INS is designed for precise measurement of the internal or/and external track geometry in mobile surveying mode, and can be adjusted for a variety of surveying tasks for different type of tracks with different optional configurations including basic configuration, GNSS/INS configuration, and TS/INS configuration. TGMT based on aided INS with any configuration can measure the gauge, cross level and twist with 0.3 mm accuracy. The differences between these three configurations and their possible application scenarios are discussed below.

TGMT based on aided INS with basic configuration is mainly designed for relative surveying scenarios, such as measuring part or all of the internal track geometric parameters, inspecting track geometric quality for different kinds of rails including high speed railway slab track, norm speed line ballast track, metro track, and tramway track in both the track construction and maintenance phase. TGMT with basic configuration is not able to provide precise 3D position coordinates of the track axis/centerline. This configuration can be operated at a very high speed up to 18 km/h.

TGMT with GNSS/INS configuration is an enhanced version of basic configuration and provides global position coordinates with centimeter absolute accuracy and submillimeter relative accuracy in measuring internal track geometric parameters including the track deformation, track irregularities together with gauge, cross level and twist. TGMT with this configuration can also be used for track geographic information system (GIS), redesign of the track, recovering the geometry axis of the existing line, documentation of GIS for building a new line besides the existing line. TGMT based on aided INS with GNSS/INS configuration can be operated at a very high speed up to 18 km/h. Introducing the carrier-phase-dominated differential GNSS combined with INS restricts its application scenario to: absolute surveying requires centimeter level 3D position, or the case which focuses on relative measurement accuracy such as track irregularity measurement which can be derived from the accurate attitude angle measurements. Attention should be paid to the GNSS signal interference and availability in the complicated railway track surveying environment especially in the tunnel.

TGMT with TS/INS configuration is designed for absolute mobile/kinematic track geometry surveying and measuring all kind of internal and external track geometric parameters for different type of track. It is able to provide 1.4 mm absolute positioning accuracy relative to the track geodetic control network together with other geometric parameters, and can be applied for railway track construction, precise adjustment, track geometric quality inspection, precise track geometry surveying and produce the adjustment data needed by the tamping machine. TGMT with TS/INS configuration can be operated at a speed of 2~3 km/h currently when the total station measurement is carried out about every 120 m, which is much slower than other two configurations due to the free stationing of the total station. Nevertheless, it is about 20 times faster than the conventional TGMTs combined with total station alone surveying in the stop-and-go mode.

## 6. Field Tests and Performance Analysis

### 6.1. GNSS/INS Configuration

#### 6.1.1. Experiment Description

**Figure 13:**
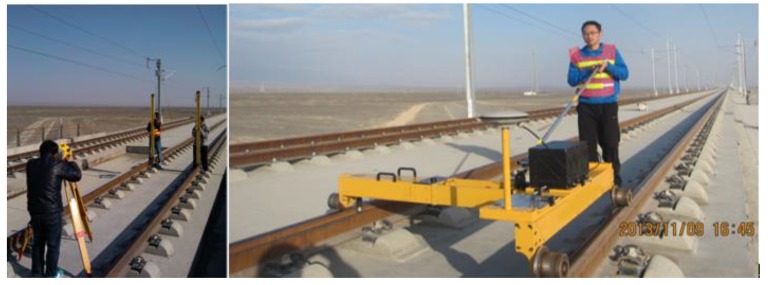
Experimental setups.

The TGMT based on GNSS/INS configuration for railway track irregularities measurement was evaluated for the first time in November 2013 on the newly built Lanzhou-Urumqi high speed railway line. Experiments were conducted to assess its performance and track irregularity surveying accuracy, i.e., relative measurement accuracy. The Lanzhou-Urumqi high speed line adopting the slab track system is a high speed passenger railway in northwestern China running from Lanzhou to Urumqi with a designed operation speed of 250 km/h. The slab track was under the precise adjustment phase for the second time when the experiment was conducted. About a 1000-m-length track section was surveyed using the TGMT based on aided INS with GNSS/INS configuration as shown in Figure 13. The trolley system depicted in the figure is the first version prototype of the TGMT based on aided INS presented in this paper.

A navigation grade INS/GNSS integrated system, integrating high precision ring laser gyros (RLG) and high stability quartz accelerometers based IMU with a professional NovAtel GNSS OEM6 card. The INS/GNSS system has almost the same performance and accuracy as the POS830 mentioned before, whose specifications are listed in Table 1. A GNSS base station sampling simultaneously was set nearby for post positioning processing in the carrier phase differential GNSS mode to provide accurate update to the inertial navigation system. The master station and rover GNSS receivers are under favorable observation conditions and sampled at 1 Hz. In this experiment, we surveyed the same track section three times with the TGMT pushed by human force in both forward and backward directions for repeatability analysis [28].

To quantitatively assess the surveying accuracy of the TGMT, we surveyed the same track section using high precision digital level and conventional TGMT based on total station only to create the surveying references. A Trimble DiNi digital level was used to measure the heights of both rails at each sleeper point with a 0.625 distance interval. The height measurement sequences by the accurate digital level is known to have a relative accuracy of 0.3 mm after errors adjustment, which is accurate enough to characterize the vertical profile of the rails and to be used as reference to evaluate the surveying accuracy of the aided INS in vertical direction. Mileage information is attached to the leveling measurement. A conventional Ambeg GRP1000 system was used as the reference system to provide desired solutions in horizontal or alignment direction. The Amberg GRP1000 system combined with high precision Leica TCA 2003 total station, is able to provide the track position determination with the accuracy of 1.4 mm in the stop-and-go mode.

#### 6.1.2. Results Analysis

The performance of the TGMT based on A-INS is assessed through both the repeatability analysis and evaluating its measurement consistency with the reference system. As mentioned above, the TGMT with GNSS/INS configuration has high relative accuracy in measuring the inner geometric parameters. The inner consistency in track geometry or track irregularity is indicated as a series of versine difference of two measurement points with specific distance interval. The parameters representing horizontal and vertical track irregularity are obtained by calculating the difference in versince of any two measurement points with a specific distance interval along the track for a chord with given length (30 m chord is predefined for the short wavelength track irregularity, and 300 m for the long wavelength) and then subtracting the related nominal value of the difference [11].

**Figure 14:**
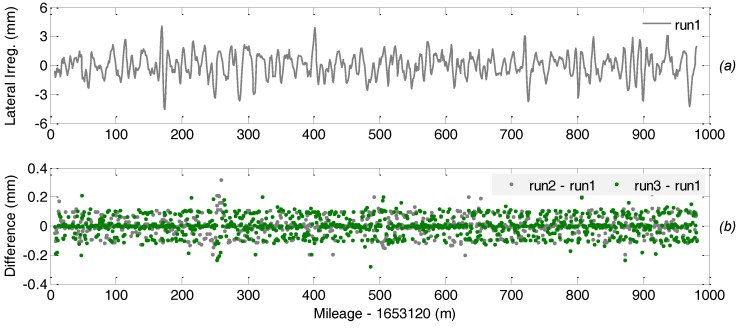
Short wavelength lateral irregularity measurement repeatability of reference rail: (**a**) measurement of the 1st run; (**b**) difference of measurement of the 2nd and the 3rd runs with respect to the 1st run.

**Figure 15:**
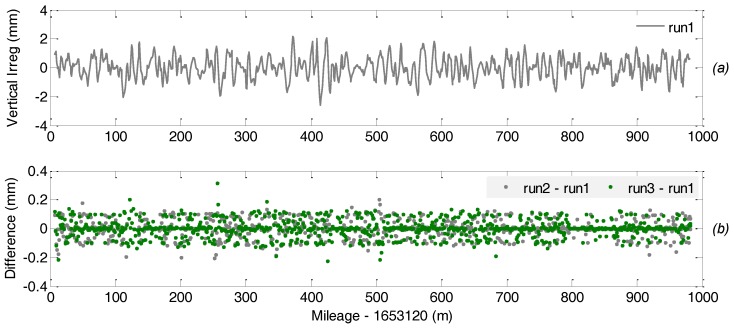
Short wavelength vertical irregularity measurement repeatability of reference rail: (**a**) measurement of the 1st run; (**b**) difference of measurement of the 2nd and the 3rd runs with respect to the 1st run.

Figure 14 and Figure 15 depict the short wavelength lateral/horizontal and vertical track irregularity measurements and the difference in measurement between multiple runs over the same track section, where the subplot (a) in each figure shows the measurement of related track geometric parameter in the 1st run and the subplot (b) shows the difference of the related measurement of the 2nd and the 3rd runs with respect to the 1st run to indicate the measurement repeatability. Comparisons show that the track irregularity measurement repeatability of the TGMT based on aided INS with GNSS/INS configuration in both horizontal and vertical direction is smaller than 0.3 mm.

**Figure 16:**
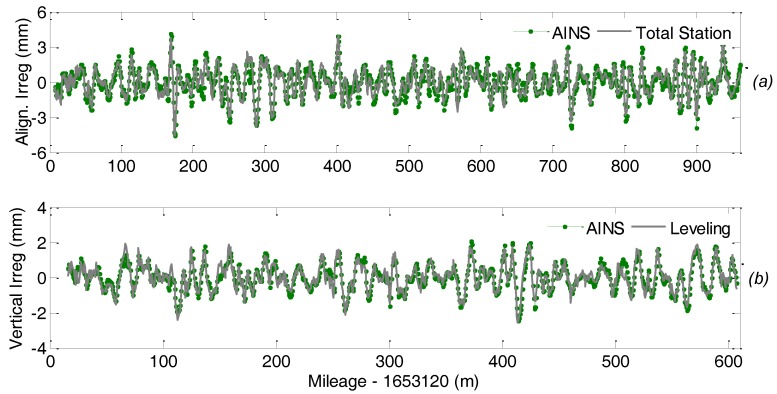
Comparison of short wavelength alignment (**a**) and longitudinal (**b**) track irregularity parameters (i.e., differential ver-sines) over 30 m distance measured by aided INS and reference system; the tolerance for short wavelength longitudinal track irregularity is 2 mm.

**Figure 17:**
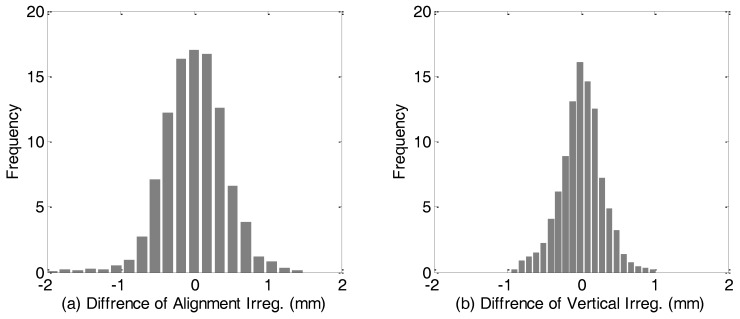
Histogram of difference of the irregularity measurements from the TGMT based on TS/INS and the reference system in horizontal (**a**) and vertical (**b**) direction, respectively.

Figure 16 depicts the comparison of short wavelength track irregularity measurement between the TGMT with GNSS/INS configuration and the reference system in both horizontal and vertical directions. The statistical values of the difference with respect to the reference mearing system is depicted in Figure 17, which shows the difference is smaller than 1 mm in vertical direction with respect to the leveling elevations and mostly smaller than 1 mm in horizontal direction with respect to the conventional TGMT measurement. The difference in horizontal direction is slightly larger than that in vertical direction, which can be explained as the reference measurement by conventional TGMT is not as accurate as the leveling and on the other hand our result has slightly lower precision in horizontal direction due to the larger heading angle measurement uncertainty. The results indicate that proposed method can achieve millimeter and even submillimeter relative accuracy in the railway track inner geometry survey and comply with the accuracy requirements specified by the normative standard for TGMT [11,28]. The proposed TGMT based on aided INS with GNSS/INS configuration has been successfully applied in several real projects, including the track precision adjustment in the new Lanzhou-Urumqi high speed railway in 2013 and the track geometric quality inspection for the Wuhan modern tramway in 2016.

### 6.2. Total Station/INS Configuration

#### 6.2.1. Experiment Description

Here we present some results of the TGMT with TS/INS configuration from a real surveying project which was carried out in October 2017 to survey the railway track geometry of the existing Beijing-Shanghai high speed railway to illustrate the measurement accuracy of the TGMT. The Beijing-Shanghai high speed railway adopting ballastless slab track system is the first one in China designed for a maximum speed of 380 km/h in commercial operations, and opened for commercial service in June, 2011. This project is to survey the absolute/external geometric parameters of the track for regular maintenance. It is required to achieve 1~2 mm position accuracy relative to the track control network so to evaluate the deterioration of the track relative to its designed position and geometry. On the other hand, it requires to survey as fast as possible since the time range permitted for surveying is limited to only about 3–4 h every day.

In this project the TGMT with TS/INS configuration is adopted for measuring the absolute 3D position and other track geometric parameters. Prior to real surveying application, experiments have been carried out to assess the measurement accuracy and performance of our TGMT by comparing with the result from an Amberg GRP1000 system. In this experiment, about 1000-m-length track was surveyed by both our trolley and the Amberg GRP1000 which is used as the reference system. The TGMT with TS/INS configuration and the reference trolley stopped about every 120 m to free stationing to obtain the absolute 3D position coordinates in the control network frame, and surveyed in kinematic mode between every two stationing points.

#### 6.2.2. Results Analysis

**Figure 18:**
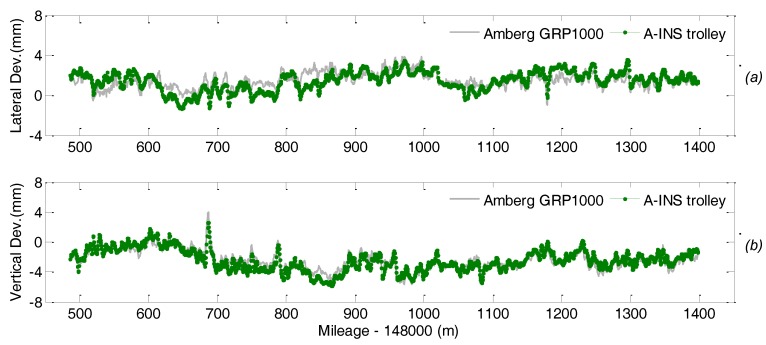
Comparison of the displacement measurements from the TGMT based on TS/INS and the reference system. (**a**) lateral displacement measurement; (**b**) vertical displacement measurement.

**Figure 19:**
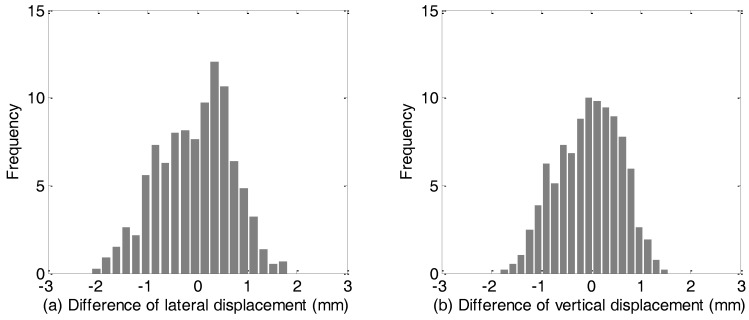
Histogram of difference of the lateral (**a**) and vertical (**b**) displacement measurements from the TGMT based on TS/INS and the reference system.

**Figure 20:**
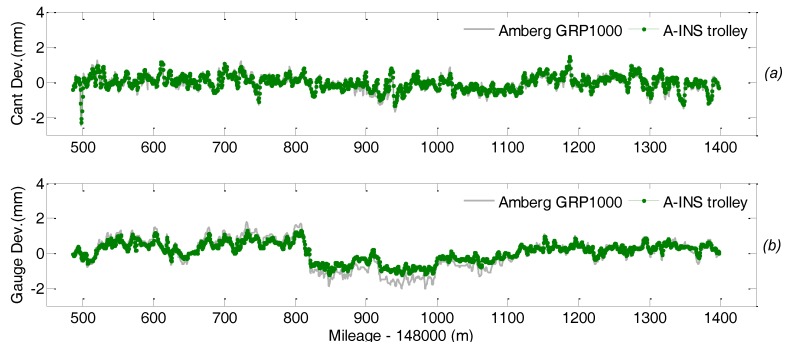
Comparison of the cross level (**a**) and gauge (**b**) measurements from the TGMT based on TS/INS and the reference system.

**Figure 21:**
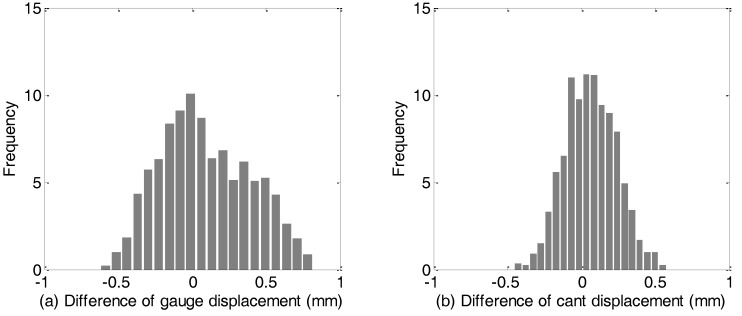
Histogram of difference of the cross level (**a**) and gauge (**b**) measurements from the TGMT based on TS/INS and the reference system.

Figure 18 depicts the comparison of the lateral and vertical displacement measurements, i.e., the absolute deviation of the track with respect to its designed positing in horizontal and vertical direction respectively, from the TGMT with TS/INS configuration and the reference system. The difference between the measurements from these two system is calculated, and a representation of whose distribution is plotted in Figure 19. Statistics shows the difference is smaller than 2 mm in both the horizontal and vertical directions. Analogous analysis is carried out for the gauge and cross level measurements and shown in Figure 20 and Figure 21. Taking into account the measuring uncertainty of the reference TGMT we can conclude that the proposed TGMT based on TS/INS can achieve about 1.4 mm accuracy in absolute 3D position coordinates surveying and 0.3 mm for the gauge and cross level measurement when involving a free stationing every 120 m.

## 7. Conclusions

We propose a modular railway track geometry measuring trolley system based on aided INS. Concepts and key points on the design and implementation of the TGMT’s architecture and the data processing of the TGTM based on aided INS with different configurations are presented in great details, which would benefit researchers in this field and those who want to implement this kind of TGMT. The surveying performance of our TGMT based on aided INS with different configurations was assessed in the real track geometry surveying experiments and projects. Results show the measurement accuracy conforms to the requirement of the normative standard. In addition, the new method proposed in this paper improves the surveying efficiency by orders of magnitude, e.g., at least 20-times faster, compared to conventional methods and TGMTs. The TGMT based on aided INS can indeed realize precise mobile/kinematic surveying of railway track geometry.

## Figures and Tables

**Table 1:** Specification of the IMU in POS830.

**Physical**			
Weight		8.5 kg	
Size		190 cm × 190 cm × 183 cm height	
Temperature range (operating)		−40 °C~+71 °C	
Power		18–32 V DC, <60 w	
**IMU**			
Parameter	Gyroscope		Accelerometers
Range	±300 deg/s		±10 g
Bias	0.01 deg/h		25 mGal
Scale factor	10 ppm		10 ppm
Sampling rate	200 Hz		
Shock	40 g		
**GNSS**			
Dual frequency, Sampling rate: 1 Hz, Position accuracy: 2 cm + 1 ppm (RMS) in RT-2 LITE mode			
